# Mosquito Immunity against Arboviruses

**DOI:** 10.3390/v6114479

**Published:** 2014-11-19

**Authors:** Shuzhen Sim, Natapong Jupatanakul, George Dimopoulos

**Affiliations:** 1 Genome Institute of Singapore, 60 Biopolis Street, #02-01 Genome, Singapore 138672, Singapore; E-Mail: shuzhens@gis.a-star.edu.sg; 2 W. Harry Feinstone Department of Molecular Microbiology and Immunology, Bloomberg School of Public Health, Johns Hopkins University, 615 N. Wolfe Street, Baltimore, MD 21205, USA; E-Mail: njupata1@jhu.edu

**Keywords:** mosquito, arbovirus, innate immunity

## Abstract

Arthropod-borne viruses (arboviruses) pose a significant threat to global health, causing human disease with increasing geographic range and severity. The recent availability of the genome sequences of medically important mosquito species has kick-started investigations into the molecular basis of how mosquito vectors control arbovirus infection. Here, we discuss recent findings concerning the role of the mosquito immune system in antiviral defense, interactions between arboviruses and fundamental cellular processes such as apoptosis and autophagy, and arboviral suppression of mosquito defense mechanisms. This knowledge provides insights into co-evolutionary processes between vector and virus and also lays the groundwork for the development of novel arbovirus control strategies that target the mosquito vector.

## 1. Introduction

Despite decades of vector control efforts, arthropod-borne viruses (arboviruses) remain a significant public health threat in large regions of the world. Dengue virus (DENV, family *Flaviviridae*) causes an estimated 400 million infections annually, with 3.6 billion people living in areas at risk for epidemic transmission [1]. The rapid spread of West Nile virus (WNV, family *Flaviviridae*) across North America [2] and the emergence of chikungunya virus (CHIKV, family *Togaviridae*) in the Indian Ocean, Europe, the Caribbean, and Central and South America [3] further illustrate the increasing severity and geographical range of arboviral diseases.

In nature, arboviruses are primarily maintained in a horizontal transmission cycle between blood-feeding arthropod vectors and vertebrate hosts. Vertical transmission of arboviruses from infected female mosquitoes to their offspring has also been reported in the laboratory and in the field (reviewed in [4]), but is generally considered to occur extremely infrequently. Arbovirus tropisms in the mosquito vector have been extensively studied in the case of DENV and *Aedes aegypti* [5]. Once ingested through a mosquito’s blood meal from an infected human, DENV first infects and replicates in the insect’s midgut epithelium. It subsequently disseminates through the hemolymph to other organs such as the fat body and trachea, finally infecting the salivary glands. Here, the virus is secreted into mosquito saliva, and injected into a human host when the mosquito next takes a blood meal [5]. These tropisms are broadly similar across other mosquito-arbovirus pairings.

Conventional vector control methods such as insecticide spraying and the removal of mosquito breeding sites have in many cases proven to be unsustainable solutions for a variety of reasons, including a lack of public awareness, adequate funds, and field training [6], as well as the development of insecticide resistance [7]. In addition, vectors such as *Ae. aegypti* are extremely well adapted to urban environments, laying their eggs in clean water in artificial containers, and displaying a preference for staying indoors.

The recent availability of draft genome sequences for medically important mosquito species such as *Ae. aegypti* [8], *Culex quinquefasciatus* [9], and *Anopheles gambiae* [10] has greatly facilitated research efforts toward understanding the functional interactions between virus and vector, laying the groundwork for the development of molecular entomological vector control strategies. Here, we review current knowledge on arbovirus-mosquito interactions, with a special focus on mosquito anti-DENV immunity.

## 2. Mosquito Antiviral Defense Pathways

**Figure 1:**
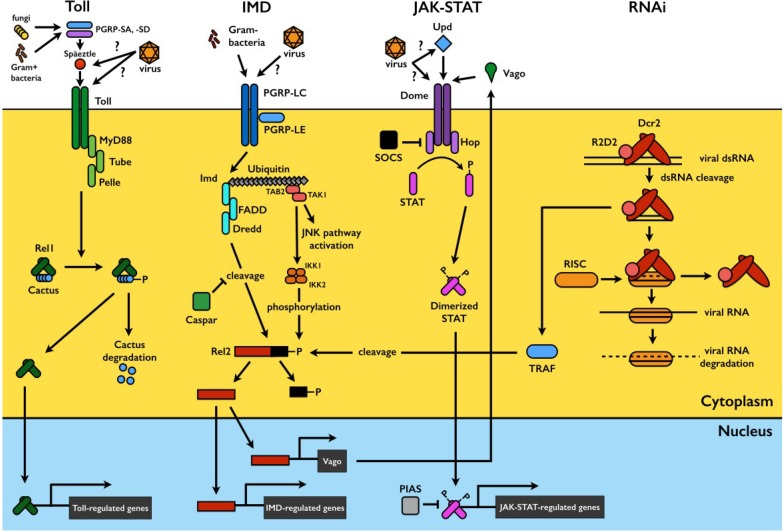
Mosquito immune signaling and RNAi pathways. In Toll pathway signaling, detection of pathogen-derived ligands by pattern recognition receptors (PRRs) such as PGRP-SA and -SD triggers proteolytic cleavage of the cytokine Späetzle, which binds to and activates the Toll receptor. This triggers signaling through the adaptor proteins MyD88, Tube, and Pelle, resulting in the phosphorylation and degradation of Cactus, a negative regulator which binds to and sequesters the Rel1 transcription factor in the cytoplasm. Cactus degradation allows Rel1 translocation to the nucleus to activate transcription of Toll-pathway regulated genes. The IMD pathway is activated by ligand binding to PGRP-LCs and -LEs. This triggers signaling through IMD and various caspases and kinases, leading to a functional split in the pathway. One branch triggers JNK signaling to activate the transcription factor AP1, while the other results in the phosphorylation of the Rel2 transcription factor and its subsequent DREDD-mediated cleavage. Activated Rel2 translocates to the nucleus to activate IMD-regulated transcription. The JAK-STAT pathway is triggered by Unpaired (Upd) binding to the receptor Dome, activating the receptor-associated Hop Janus kinases, which phosphorylate each other and subsequently recruit and phosphorylate the STAT transcription factor. Phosphorylated STATs dimerize and translocate to the nucleus to activate JAK-STAT-regulated transcription. The exogenous siRNA pathway is activated when virus-derived long dsRNA is recognized and cleaved by Dcr2 into siRNAs, usually 21 bp in length. siRNAs are loaded onto the multi-protein RISC complex, which degrades one strand of the duplex and uses the other for targeted degradation of complementary single stranded viral RNA. Sensing of viral dsRNA by Dcr2 also activates TRAF, leading to Rel2 cleavage and activation via a distinct pathway. Rel2 activates transcription of Vago, a secreted peptide which subsequently triggers JAK-STAT pathway signaling. Please refer to the text for more details.

Mosquitoes are exposed to a wide variety of microorganisms in their habitats and during sugar and blood feeding. The mosquito’s innate immune system mounts a potent immune response against microbial challenge and is capable of distinguishing among broad classes of microorganisms (reviewed in [11,12]). In this section, we focus on the major mosquito immune signaling pathways that have been implicated in the antiviral defense, namely the Toll, immune deficiency (IMD), and Janus kinase/signal transducers and activators of transcription (JAK-STAT) pathways. In addition, we will consider the RNA interference (RNAi) pathway; though not a classical innate immune pathway, it also plays a key role in antiviral defense. A visual summary of each pathway is presented in Figure 1.

### 2.1. The Toll Pathway

The Toll pathway was first characterized in *Drosophila melanogaster* in the context of its role in embryonic development and was later found to play a crucial role in the fly’s defense against fungi, Gram-positive bacteria, and viruses [13,14,15]. Toll pathway signal transduction is very similar to mammalian NF-kB signaling: Recognition of pathogen-derived ligands by pattern recognition receptors (PRRs) such as peptidoglycan recognition proteins (PGRP)-SA and -SD [16,17] activates a proteolytic cascade that leads to cleavage of the cytokine Späetzle [18], a cysteine knot molecule with structural similarities to mammalian neurotrophins. Späetzle binds to and activates the Toll transmembrane receptor [19], triggering signaling through the associated adaptor proteins MyD88 and Tube and the kinase Pelle. This activation results in the phosphorylation and subsequent proteasomal degradation of the negative regulator Cactus [20,21], which binds to and sequesters the NF-kB-like transcription factor Dorsal (Rel1 in mosquitoes) in the cytoplasm. Cactus degradation allows Dorsal/Rel1 translocation to the nucleus and subsequent transcription of effector genes such as antimicrobial peptides (AMPs) [12,22] (Figure 1).

The Toll pathway is conserved in mosquitoes and also plays a key role in antiviral defense in these insects. DENV infection of the *Ae. aegypti* midgut, carcass, and salivary gland activates the transcription of Toll pathway components and putative effectors such as Späetzle, Toll, Rel1A, and multiple AMPs [23,24,25]. The DENV-infected mosquito transcriptome and that of Cactus-silenced (or Rel1-activated) mosquitoes also overlap considerably in terms of the magnitude and direction of gene regulation [23].

Functional assays provide further evidence for the role of the Toll pathway in DENV control: Transient activation of Rel1 through RNAi-mediated gene silencing of Cactus significantly reduces midgut DENV titers, whereas silencing of the adaptor protein MyD88 leads to significantly increased DENV titers [23]. The Toll pathway controls DENV replication in the midgut as early as 3 days post-infection, and the Toll pathway-regulated defense is active against multiple DENV serotypes as well as in field-derived mosquitoes [26,27].

Stable transinfection of *Ae. aegypti* with the endosymbiont bacterium *Wolbachia* greatly limits infection of the mosquito vector with a range of human pathogens, including DENV and CHIKV [28,29,30,31]. This stable transinfection can occur via several mechanisms (reviewed in [32]), one of which is the induction by *Wolbachia* of reactive oxygen species (ROS) production by the mosquito, resulting in Toll pathway activation and the subsequent production of the AMPs cecropin and defensin, which hinder DENV replication [33].

Sindbis virus (SINV, family *Togaviridae*) has been reported to induce the transcription of Dif, a mosquito Toll pathway-activated transcription factor, during early-stage infection in *Ae. aegypti* [34]. The role of the Toll pathway in anti-WNV defense is unclear: WNV infection of *Cx. quinquefasciatus* does not significantly alter the expression of Toll pathway components or effectors [35], while infection of *Ae. aegypti* has been reported to down-regulate the mosquito ortholog of *Drosophila* Späetzle 5 [36].

### 2.2. The IMD Pathway

The immune deficiency (IMD) pathway is well known to play crucial roles in insect defense against bacteria [37,38,39]. In *Drosophila,* activation of the IMD pathway, like that of the Toll pathway, is initiated by PRR-mediated recognition of microbial pathogen-associated molecular patterns (PAMPs) (reviewed in [40]). Intracellular signaling through the adaptor IMD protein and various caspase-like proteins and kinases then leads to a functional split in the pathway into two downstream branches [37,41,42,43]. One branch, similar to the mammalian c-Jun/JNK pathway, activates the transcription factor AP-1 via JNK signaling [44,45], while the other branch culminates in the processing and activation of the NF-kB transcription factor Relish (Rel2 in mosquitoes) via caspase-mediated cleavage of its carboxy-terminal end [38,39]. Activated Relish is then translocated to the nucleus to promote the transcription of anti-microbial effectors [46,47]. The human Fas-associated factor 1 ortholog Caspar negatively regulates Relish activation, possibly by interfering with the enzymes involved in its cleavage [48] (Figure 1). In mosquitoes, the IMD pathway also plays important roles in the antibacterial defense, and it also directs immune responses against *Plasmodium* parasites [49,50,51,52].

The antiviral role of the IMD pathway has more recently been investigated, and in flies it has been found to be active against SINV and cricket paralysis virus (CrPV) [53,54]. In mosquitoes, up-regulation of IMD components and effectors in response to DENV and SINV infection has been observed [24,34], but transient activation of the pathway by RNAi-mediated gene silencing of Caspar has no effect on mosquito midgut DENV titers [23]. A more recent study, however, has found thatcompromising the pathway in DENV-refractory strains of *Ae. aegypti* via IMD silencing results in increased midgut DENV titers [27], suggesting that the IMD pathway may be required for anti-DENV defense, but that in susceptible strains it may already be operating at maximum capacity. The situation may be different during Semliki Forest Virus (SFV, family *Togaviridae)* infection of *Ae. albopictus* cells, however, where the addition of heat-inactivated Gram-negative bacteria (which stimulates the IMD and JAK-STAT pathways simultaneously) prior to virus challenge results in reduced SFV replication [55].

### 2.3. The JAK-STAT Pathway

The Janus kinase/signal transducers and activators of transcription (JAK-STAT) pathway was first identified as an interferon (IFN)-induced signaling pathway in vertebrates [56,57], and it plays a key role in antiviral immunity in mammals [58,59]. It is conserved in invertebrates and was first characterized in *Drosophila* in the context of its role in several aspects of development (reviewed in [60]).

The canonical *Drosophila* JAK-STAT pathway is triggered by the attachment of the Unpaired (Upd) peptide ligand to the extracellular region of the transmembrane receptor Dome. Ligand recognition leads to conformational modification and dimerization of Dome, resulting in self-phosphorylation of the receptor-associated Janus kinases (JAKs). Activated JAKs then phosphorylate the cytoplasmic tail of the receptor, generating docking sites for the recruitment of STAT proteins. The recruited STATs are then phosphorylated by the Dome/JAK activated complex, resulting in STAT activation and dimerization. The activated STAT dimers are translocated to the nucleus and induce the expression of effector genes [60,61] (Figure 1).

The first evidence for JAK-STAT pathway involvement in insect immunity came from studies in the malaria vector mosquito *An. gambiae*, in which bacterial challenge resulted in nuclear translocation of STAT [62]. It was later found that the JAK-STAT pathway modulates viral infection and survival of flies infected with the *Drosophila* C virus (DCV), suggesting an evolutionarily conserved antiviral mechanism in insects and humans [63].

The antiviral role of the JAK-STAT pathway is conserved in the *Ae. aegypti* defense against DENV. DENV replication in the mosquito midgut is significantly increased when the pathway is transiently suppressed by RNAi-mediated depletion of the receptor Dome or the JAK ortholog Hop, and the opposite effect on virus replication is observed when the pathway is activated by silencing of protein inhibitor of activated STAT (PIAS), a negative regulator [64]. As mentioned above, prior stimulation of the IMD / JAK-STAT pathways with Gram-negative bacteria reduces SFV replication in *Ae. albopictus* cells [55], but it is difficult to distinguish between the roles of these two pathways in this case.

JAK-STAT pathway-activated antiviral mechanisms are poorly understood. Two DENV-induced, JAK-STAT-regulated putative effector genes that restrict DENV replication in midgut tissues have been identified but remain uncharacterized [64]. These were termed dengue virus restriction factors (DVRFs) 1 and 2: DVRF1 is a putative transmembrane protein that presumably acts as a pathway receptor; DVRF2 contains antifreeze and allergen domains and may be involved in virus recognition. Immunity-related genes comprise only a small proportion of the mosquito’s JAK-STAT-regulated transcriptome, suggesting that this pathway may not fight DENV infection through a classical innate immune response [64].

In contrast to *Aedes* and *Culex* mosquitoes, which transmit many arboviruses, *Anopheles* mosquitoes are known to be the primary vector for only one—O’nyong-nyong virus (ONNV, family *Togaviridae)*. Differences between the immune responses of culicine and anopheline mosquitoes lead to one hypothesis for this distinction. Intriguingly, ONNV infection of *An. gambiae* does not appear to transcriptionally activate the Toll, IMD, or JAK-STAT pathways, and knockdown of Toll or JAK-STAT pathway components also had no effect on ONNV replication [65]. In this study, however, mosquitoes were intrathoracically inoculated with ONNV instead of orally infected [65], and so it remains possible that these immune pathways are active against ONNV in the midgut, while other responses target it upon dissemination. Indeed, several ONNV antagonists have been identified, including an MD2 co-receptor-like gene, a galectin, two lysozymes [65], and a heat-shock protein [66,67,68]. The mechanisms by which these gene products mediate virus killing remain unknown, but may involve processes such as immune recognition, immune signaling, membrane perturbation, and modulation of cellular stress responses. ONNV infection also activates the transcription of numerous LRIMs and thioester-containing proteins (TEPs), suggesting that complement-mediated lysis or opsonization may also act against virus [65].

### 2.4. The RNA Interference (RNAi) Pathway

The RNAi antiviral mechanism is not a classical pathogen-stimulated immune response, but instead appears to use enzymes that are constitutively expressed in the cell cytoplasm [23,34]. The insect exogenous small interfering RNA (siRNA) response is the best studied of the RNAi pathways, and is initiated when long, virus-derived double-stranded RNA (dsRNA) in the cytoplasm of infected cells is recognized and cleaved by Dicer-2 (Dcr2) into siRNAs, usually 21 base pairs (bp) in length. siRNAs are then loaded onto the multi-protein RNA-induced silencing complex (RISC), which unwinds the duplex RNA and degrades one of the siRNA strands, using the other for targeted degradation of single-stranded viral RNA with sequence complementary to the siRNA (reviewed in [69]) (Figure 1).

RNAi had earlier been shown to influence RNA virus replication and pathogenic outcomes in *D. melanogaster* [70,71] but has only more recently been recognized as an antiviral mechanism in mosquitoes. Studies in transgenic mosquito lines with inducible midgut and salivary gland expression of DENV-specific dsRNA have shown that RNAi inhibits DENV infection in these organs [72,73]. The use of dsRNA to transiently deplete key RNAi components has provided direct evidence for a natural antiviral role of RNAi in *Ae. aegypti*: Knockdown of the effector enzymes Dcr2, R2D2, and Argonaute-2 allows DENV to replicate more efficiently after oral challenge. Knockdown of these components also shortens the extrinsic incubation period of DENV in mosquitoes and increases the efficiency of virus transmission, suggesting that RNAi is a key mediator of vector competence [74]. RNAi has also been found to control SINV and CHIKV replication in *Ae. aegypti* [75,76]. It is also a key component of anti-ONNV defense in *An. gambiae* [77,78], in which the Toll, IMD, and JAK-STAT pathways do not appear to play antiviral roles during systemic infection [65].

Arbovirus infection of mosquitoes is generally asymptomatic and persists for the life of the vector, but the virus appears to be continually targeted by the RNAi response. DENV2 and WNV siRNAs are detected at both 7 and 14 days after oral infection, and more siRNAs accumulate at later times after infection [74,79]. Interestingly, DENV2-derived small RNAs make up less than 0.05% of the total small RNAs sequenced, a proportion similar to that observed for West Nile virus (WNV) in *Culex* mosquitoes [79] but much lower than that observed for alphaviruses (10% for SINV) [78]. It has been suggested that sequestration of flavivirus replication complexes in membrane-enclosed vesicles limits the access of Dcr2 to dsRNA replicative intermediates, and that given the low abundance of DENV2-specific small RNAs, Dcr2 cleavage of dsRNA alone may be sufficient to control DENV infection [80].

Although the origin of the initiating dsRNA for RNAi activation is not yet fully defined in mosquitoes, replicative intermediates formed between the positive and negative viral RNA strands or secondary structure within an individual viral RNA molecule are likely sources; these two possibilities are not mutually exclusive. Deep sequencing of small RNAs from DENV-infected mosquitoes has revealed an almost equal ratio of positive- to negative-sense DENV-derived small RNAs [80], suggesting that most small RNAs are derived from dsRNA replicative intermediates instead of intra-strand secondary structures.

While 21-bp virus-derived siRNAs are the dominant species during the middle and late stages of DENV infection, another study found a predominance of longer (24–30-bp) DENV2-derived small RNAs during early infection [81]. These RNAs are most likely derived from the PIWI RNA (piRNA) pathway, suggesting a role for this Dcr2-independent pathway in antiviral defense [81]. Virus-derived piRNAs have also been detected during CHIKV infection of *Ae. aegypti and Ae. albopictus* [82]. SFV infection of mosquito cell lines also triggered virus-derived piRNA production, and knockdown of piRNA pathway components enhanced SFV infection in these cells, further implicating this small RNA pathway in antiviral defense [83].

Studies in flies may provide further insight into the antiviral mechanisms of RNAi. Systemic spread of RNAi via cellular dsRNA uptake has been reported to be necessary for *D. melanogaster* survival after RNA virus challenge [84]. Since the flies in this study were challenged via septic injury with a pathogenic virus (DCV) and a virus that does not typically infect drosophilids (SINV) [84], lysis of infected cells may have released dsRNA for uptake by other cells. Although the spread of RNAi has been described in cultured mosquito cells infected with SFV [85], it is unclear whether systemic RNAi is necessary for the mosquito defense against arboviral infections, which have classically been regarded as non-pathogenic in the vector.

Another study from the *Drosophila* model suggests a role for insect-encoded reverse transcriptase (RT) enzymes and the RNAi machinery in maintaining the persistence of RNA viruses. Here, viral genome fragments are reverse-transcribed and inserted into the insect genome by retrotransposon elements; these insertions later serve as templates for RNAi responses against the virus [86]. Given that the *Ae. aegypti* genome also contains RTs and transposable elements [87], and that flavivirus and rhabdovirus sequence fragments have been detected in the genomes of *Aedes* species [88,89], it would be intriguing to study this phenomenon in mosquitoes.

### 2.5. Pathway Crosstalk

The Toll and IMD pathways have been reported to dually regulate subsets of immune effector genes [50,90,91], and there is growing evidence for a functional overlap between insect antiviral, anti-bacterial, and anti-parasite immune responses [23,49,92]. Although it is still poorly understood, crosstalk between pathways is likely to occur so that an effective immune response can be coordinated.

The best-studied example of pathway crosstalk in insect antiviral immunity involves Dcr2, the RNAi pathway DExD/H-box helicase that recognizes viral dsRNA and cleaves it into siRNAs. In addition to its role in RNAi, Dcr2 has been reported to mediate the induction of antiviral genes, similar to the action of mammalian cytoplasmic RIG-I-like dsRNA sensors: DCV infection of *Drosophila* induces the expression of the antiviral polypeptide *Vago* in a Dcr2-dependent manner [93]. Other components of the RNAi machinery do not appear to be involved in the modulation of *Vago* gene expression, suggesting that its induction through Dcr2 is independent of the RNAi pathway [93].

Recently, this mechanism has also been characterized in the *Cx. quinquefasciatus* response to WNV. Here, sensing of viral dsRNA by Dcr2 activates TNF receptor-associated factor (TRAF), which in turn leads to Rel2 cleavage and activation. Activated Rel2 binds to a conserved NF-kB binding site on the *Cx*Vago promoter, inducing its transcription [94,95]. *Cx*Vago, a secreted peptide, restricts WNV by activating the JAK-STAT pathway [94] (Figure 1). These findings are in contrast to those of the *Drosophila* study, in which the induction of fly *Vago* was not mediated by NF-kB transcription factors, and *Vago* did not activate the fly JAK-STAT pathway [93]. Interestingly, the *Aedes* ortholog of *Vago* was also found to restrict WNV in *Culex* cells, suggesting that the antiviral action of this molecule may be conserved in mosquito vectors of DENV [94]. These findings characterize a distinct pathway leading to Rel2 activation and suggest potential crosstalk mechanisms between the mosquito RNAi, JAK-STAT, and IMD pathways.

Other peptides with cytokine-like antiviral activity have been isolated from arbovirus-infected mosquito cells: The anionic septapeptide viprolaxikine, for example, has been isolated from the cell-free medium supernatants of DENV-infected *Ae albopictus* C6/36 cells; this septapeptide protects against DENV infection in naive mosquito and vertebrate cells [96,97]. The molecular mechanisms of this protection, however, have yet to be studied.

## 3. Arbovirus Interactions with Host Cell Processes and Host Factors

Arboviruses are obligate intracellular pathogens that exploit the host’s cellular machinery in order to replicate. The intracellular replication cycle for DENV (reviewed in [98]), for example, has been well studied and is likely to be similar in insects and vertebrates. DENV enters cells via clathrin-dependent receptor-mediated endocytosis (RME), and uncoating of the positive-strand RNA viral genome requires trafficking through an acidic endosomal compartment [99,100,101]. The receptors and proteins of the mosquito midgut that interact with the virus during early infection stages (reviewed in [102]) are poorly characterized. Translation of viral RNA (vRNA) occurs on endoplasmic reticulum (ER)-derived membranes, producing a single polypeptide that is then processed into individual structural and non-structural proteins. vRNA replication occurs through the production of a negative-strand intermediate that serves as a template for the synthesis of multiple copies of positive-sense vRNA. The structural proteins C, prM, and E are then produced in large quantities through successive rounds of translation and assembled with vRNA in the ER. Virions mature in the Golgi and exit the cell via the host’s secretory pathway.

While arboviruses have co-opted host cellular processes and factors to promote their own replication, fundamental cellular pathways such as apoptosis and autophagy have in some cases also evolved antiviral functions. These pathways, however, appear to have contrasting roles in different virus–host pairings, perhaps reflecting diverse co-evolutionary mechanisms.

### 3.1. Apoptosis

In vertebrates, apoptotic cell death was long ago suggested to be an innate immune defense mechanism against virus infection [103,104]. While arbovirus infection of insect cell lines tends to cause persistent, non-pathogenic infections, there is growing evidence for the antiviral role of apoptosis in live insect models [105].

Apoptosis of *Cx. pipiens* midguts has been observed during WNV infection, and this process has been thought to provide a mechanism for limiting disseminated infection in refractory mosquito strains [106]. Apoptotic cells in the salivary gland of WNV-infected *Cx. quinquefasciatus* have also been reported to be correlated with a lower proportion of infectious mosquitoes [107]. In *Ae. aegypti*, oral DENV infection of refractory and susceptible strains results in an up-regulation of the pro-apoptotic gene michelob_x (an ortholog of *Drosophila reaper*) only in refractory mosquitoes [105]. A separate study has found that RNAi-mediated silencing of the primary apoptotic caspase Aedronc increases infection prevalence in a refractory *Ae. aegypti* strain [108]. On the other hand, some arboviruses may use apoptosis as a means of facilitating their dissemination in mosquitoes. Activation of apoptosis by silencing the *Ae. aegypti* inhibitor of apoptosis (IAP) gene results in increased midgut SINV titers, while silencing Aedronc has the opposite effect [109].

### 3.2. Autophagy

During autophagy, cells enclose cytoplasmic components such as damaged organelles or proteins in *de novo-*synthesized double-membrane structures called autophagosomes; these structures then fuse with lysosomes to bring about degradation of their contents. Autophagy is a basic cellular process that maintains cellular homeostasis during stress or nutrient deprivation, allowing for recycling of cellular resources. During viral or bacterial infection, autophagy may activate and regulate immune responses, as well as directly eliminate intracellular microbes (reviewed in [110]).

A protective role for autophagy has been reported in the *Drosophila* defense against vesicular stomatitis virus (VSV, family *Rhabdoviridae*) [111], and more recently Rift Valley fever virus (RVFV, family *Bunyaviridae*) [112]. In both cases, the Toll-like receptor ortholog Toll-7 on the cell surface is thought to be responsible for virus recognition and for triggering autophagy via phosphatidylinositol 3-kinase (PI3K)-Akt signaling [112,113].

In other virus-host systems, however, autophagy may instead facilitate arbovirus infection. SINV infection of flies and mosquito cells has been reported to activate the PI3K-Akt pathway, and inhibiting PI3K-Akt signaling also restricts SINV replication [114]. A proviral role for autophagy has also been reported in mammalian systems. DENV, for example, triggers the formation of autophagosomes in human cell lines, and virus replication is restricted upon inhibition of the autophagy pathway [115]. The virus is thought to localize replication complexes to autophagosome membranes [116] and also to usurp the autophagy mechanism in order to regulate cellular lipid metabolism so as to promote virus replication [117]. However, the role of autophagy in DENV infection of mosquitoes remains to be determined.

### 3.3. The Vacuolar ATPase Complex

The vacuolar ATPase (vATPase) is a multisubunit enzyme located in the membranes of endosomes, lysosomes, and secretory vesicles. The vATPase complex brings about the acidification of these organelles via an ATP-dependent rotary mechanism that drives proton transport [118]. This process is important for DENV replication, since an acidic pH in the late endosome is required for DENV membrane fusion and RNA genome entry into cells [99,100]. Bafilomycin, a specific inhibitor of vATPases, has been reported to inhibit flaviviruses in both mammalian and insect cells [119,120], and a recent study found that chemical inhibition of vATPase by injecting or feeding adult *Ae. aegypti* with bafilomycin also restricts DENV replication in the midgut and salivary glands [121].

Various vATPase subunits have been found to be transcriptionally up-regulated in DENV-susceptible strains of *Ae. aegypti*, when compared to refractory strains [27,122]. In yeast, individual deletion of all of the subunit genes results in either a complete loss of assembly of the complex or an inactive vATPase [118]; in *Ae. aegypti*, RNAi-mediated silencing of each of the five subunits individually has been found to restrict DENV replication in both field-derived and laboratory *Ae. aegypti* strains [27,121]. Taken together, these pieces of evidence indicate the importance of a functional vATPase complex for DENV replication in mosquitoes, making this complex a promising target for chemical interventions such as treatment with small-molecule inhibitors of DENV replication.

### 3.4. The Myeloid Differentiation 2-Related and Niemann-Pick Type C1 Proteins

The myeloid differentiation 2-related lipid recognition (ML) and Niemann-Pick type C1 (NPC1) gene families encode proteins with diverse roles related to their lipid-binding domains. ML proteins are involved in processes such as lipid trafficking and metabolism, pheromone perception, and pathogen recognition [123,124,125]: mammalian MD2, for example, is a co-receptor for Toll-like receptor 4 (TLR4) binding to bacterial lipopolysaccharide [126,127], and silencing of *An. gambiae* AgMDL1 significantly increases midgut *Plasmodium falciparum* infection levels [128]. NPC1 proteins are involved in cholesterol transport and homeostasis in the late endosome [125] and also play roles in host–pathogen interactions. For example, Ebola virus requires mammalian NPC1 for membrane fusion and escape from the endosome [129,130].

DENV infection induces the expression of *Ae. aegypti* ML33 and NPC1b, and RNAi-mediated silencing of these two genes restricts virus infection in both laboratory and field-derived strains, suggesting a role for these family members in facilitating virus infection or replication [131]. Since DENV is an enveloped virus, it is possible that these lipid-binding proteins facilitate virus fusion and/or escape from the endosome, as is thought to be the case for Ebola virus [129,130].

Since silencing ML33 and NPC1b also induces the expression of a number of genes known to be controlled by the Toll, IMD, and JAK-STAT pathways, it is also possible that these genes facilitate DENV replication by negatively regulating the mosquito’s immune response [131].

In contrast, an ML family member appears to function as a virus antagonist during ONNV infection of *An. gambiae*. Here, knockdown of AgMDL1 (also referred to as ML1) resulted in increased ONNV replication [65]. Given the role of mammalian MD2 in TLR signaling [126,127], it is possible that AgMDL1 may recognize viral PAMPs and activate immune responses in this virus-vector combination. As yet, however, no Toll receptor binding partners for the MLs have been identified in insects, and so more evidence is required to support this hypothesis.

## 4. Arbovirus Suppression of Insect Immune Responses

A hallmark of viruses is their ability to suppress or evade host defenses. This facility has been well described for arbovirus infection in vertebrates: The DENV NS4B protein antagonizes the vertebrate IFN pathway by blocking STAT1 phosphorylation and activation [132,133], and DENV NS5 binds STAT2 and targets it for proteasomal degradation [134]. Japanese encephalitis virus (JEV, family *Flaviviridae*) inhibits STAT phosphorylation in vertebrate cells [135,136], and alphaviruses such as SINV, SFV, and Venezuelan equine encephalitis virus (VEEV, family *Togaviridae*) inhibit host cell transcription [137,138,139].

Viral suppression of mosquito immune responses has also been described, but the mechanisms by which this occurs are less well known. DENV infection of the *Ae. aegypti* cell line Aag2 results in the transcriptional down-regulation of numerous immunity-related genes and impairs the ability of the cells to produce AMPs in response to secondary challenge with bacteria [140]. Down-regulation of several AMPs at early time points in DENV infection has also been reported in live mosquitoes [26]. JEV blocks STAT phosphorylation in *Ae. albopictus* C6/36 cells [141], and SFV and CHIKV have been reported to suppress Toll, IMD, and JAK-STAT signaling in *Aedes* cell lines [55,75]. The SFV and CHIKV studies, however, used *Drosophila* promoter*-*reporter constructs to measure immune pathway activity. It is unclear if the specificity of these promoters is maintained in mosquito cells; the Toll pathway construct, for example, uses the *Drosophila Drosomycin* promoter despite the lack of the *Drosomycin* gene in the *Aedes* genome [68].

Although insect-only viruses encode potent viral suppressors of RNAi (VSRs) [142,143], the situation is less clear for arboviruses. The results of a recent study indicate that the DENV NS4B protein suppresses RNAi in mammalian and insect (Sf21, non-mosquito) cells, possibly by interfering with Dcr2 activity [144], but no other arbovirus-encoded protein VSRs have been reported. It is possible that arboviral VSRs have been selected against because they negatively affect vector fitness and hence virus transmission; this possibility is supported by the results of studies showing that infection of mosquitoes with SINV expressing the flock house virus (FHV, an insect-only virus) VSR B2 dramatically enhances viral replication but reduces mosquito lifespan [78,145]. Indeed, RNAi may contribute to the genetic diversity of arboviruses in mosquitoes and drive virus evolution [79].

All flaviviruses produce large quantities of a sub-genomic RNA derived from incomplete degradation of the 3' untranslated region (UTR) of genomic RNA by the cellular 5'-3' exonuclease XRN1 [146]. This sub-genomic flavivirus RNA (sfRNA) is required for WNV pathogenicity in cell lines and animal models [146] and has been reported to inhibit RNAi in both vertebrate and insect cells [147] as well as IFN-mediated antiviral responses in mammalian cells [148,149,150]. DENV sfRNA antagonizes the IFN response by binding to G3BP1, G3BP2, and CAPRIN1, which are required for the translation of IFN-stimulated mRNAs [150]. It remains to be seen whether sfRNAs also inhibit classical immune signaling pathways in insects.

Arboviruses can also influence vector responses by modulating insect microRNA (miRNA) expression. DENV infection of *Ae. aegypti* mosquitoes [151] and CHIKV infection of *Ae. albopictus* cells [152] results in the differential expression of several mosquito miRNAs that are predicted to regulate the expression of genes with potential roles in virus replication and dissemination. Functional assays will be required to determine whether these miRNAs mediate host defense responses or viral immune suppression mechanisms. Alternatively, the arboviral genome itself may also encode miRNAs: WNV has been reported to encode an miRNA-like small RNA in its 3' UTR that modulates mosquito gene expression and facilitates virus replication [153].

## 5. Natural Variation in Vector Competence for Arboviruses

There is wide variation in the susceptibility of mosquito populations to arboviruses [27,154,155,156]. This variation is presumably controlled by both genetic and environmental factors. Physiologically, an arbovirus must overcome several barriers in order to be transmitted: The inability of a virus to establish infection in the midgut (due to interference with receptor binding, uncoating, translation, or transcription, for example) is referred to as a midgut infection barrier (MIB), whereas the inability to disseminate to secondary organs and peripheral tissues (as the result of defects in the release of virions from midgut epithelial cells) is termed a midgut escape barrier (MEB). Salivary gland infection and escape barriers (SIB and SEB) have also been reported (reviewed in [157]).

The selection of *Ae. aegypti* strains with MIB and MEB for DENV has facilitated our understanding of the genetics of vector competence [158,159]. Susceptibility to DENV infection appears to be an additive trait under the control of multiple genetic loci [158,160]. Quantitative trait locus (QTL) mapping has identified several loci that control MIB and MEB [160,161,162], but specific genes or polymorphisms have not yet been pinpointed. The best characterized of these loci is the early trypsin locus: The addition of soybean trypsin inhibitor to a blood meal impairs DENV midgut replication and subsequent dissemination, suggesting that blood meal digestion and possibly proteolytic processing mediated by trypsin affect DENV infection [163]; however, a separate study has found no associations between segregating sites in early trypsin and DENV susceptibility in a population of *Ae. aegypti* from Mexico [164].

As is true for many other host–pathogen interactions, the vector competence of mosquitoes for arboviruses also appears to be influenced by genotype-by-genotype (GxG) interactions, in which infection and dissemination are affected by the specific combination of mosquito and virus genotypes [165,166]. Natural polymorphisms in *Ae. aegypti* Dcr2, for example, have been found to be associated with resistance to DENV infection in a virus isolate-specific manner; these data led the authors of the study to hypothesize that the association specificity may be the result of differences in the affinity of Dcr2 for particular viral dsRNA sequences [167]. GxG interactions complicate genetic mapping studies because resistance loci or alleles are likely to change, depending on the combination of mosquito population and virus strain [168].

Variation at the transcriptome level can also influence vector competence for arboviruses [27,122,169]. Diverse factors such as geographic location, passage history, nutrient limitation, sex-dependent selection, and exposure to pathogens have potential to shape the transcriptome [170,171,172,173], for example, through selection for mutations in *cis-* and *trans-* regulatory elements [174]. A study comparing the transcriptomes of a panel of *Ae. aegypti* strains from geographically distinct DENV-endemic regions found that DENV-refractory strains exhibited higher levels of numerous immunity-related transcripts than did susceptible strains, suggesting that differences in baseline immune activation affect vector competence [27]. Long-term exposure to environmental factors at their locations of origin is likely to have shaped the transcriptomes of these strains; the role of natural midgut microflora in stimulating basal immunity in mosquitoes, for example, has been well described [23,92,175], and co-evolution of each strain with unique suites of microbial species may have resulted in transcriptomic divergence. The impact of gut bacteria on vector competence for arboviruses (reviewed in [176]), while outside the scope of this article, will be a critical factor to consider when developing transmission blocking interventions.

Furthermore, while the Toll, IMD, JAK-STAT, and RNAi pathways play key antiviral roles in both laboratory and field-derived mosquitoes, it should be noted that these pathways have been found to control arbovirus infection to different extents, depending on the mosquito strain [27]. Strain-specific factors acting independently of these pathways are also likely to make important contributions.

## 6. Challenges and Future Directions

We are just beginning to appreciate the complexity of the functional interactions between arboviruses and their mosquito vectors. Although we now have strong evidence for the involvement of classical immune signaling pathways in the mosquito’s antiviral defense, the molecular mechanisms by which these pathways are activated and subsequently affect the antiviral response are still not well understood. The integration of signals from multiple pathways to coordinate the antiviral response, as in the Dcr2- and Rel2-dependent activation of mosquito Vago [94,95], is also an intriguing new area of study.

Characterizing the modes of action of these antiviral mechanisms is a challenge that will have important practical applications. Transgenic mosquito transmission-blocking strategies aim to render vectors refractory to arboviral infection via the stable introduction of a transgene; such strategies include the over-expression of immune pathway activators [177,178] or antiviral effectors [72]. A related approach is paratransgenesis, in which bacterial or fungal members of the mosquito microbiome can be engineered to express anti-pathogen molecules [179,180,181]. Currently, however, there is a dearth of characterized candidate antiviral gene products available for use in these approaches.

The characterization of arboviral interactions with basic host cell processes will also provide numerous opportunities for control strategies, such as the development of small molecule inhibitors of proviral host processes or factors. These fundamental cellular processes are complex, highly regulated, and involved in diverse aspects of insect physiology, so it is perhaps not surprising that they appear to play antiviral roles in some vector-virus combinations and proviral roles in others.

Field applications of novel control strategies targeting the mosquito vector may be complicated by genetic and transcriptomic divergence in mosquito and virus strains, as well as by environmental factors such as varying suites of mosquito midgut microbiota. A combination of high-throughput functional genomics, a detailed molecular understanding of virus–vector interactions, and extensive field testing will most likely be required to overcome these challenges.
